# English and American Contract Surgeons

**Published:** 1900-01

**Authors:** 


### ENGLISH AND AMERICAN CONTRACT SURGEONS.

ACCORDING to newspaper and medical journal reports the United States is not the only country which finds itself short of medical officers in time of war. We all know how necessary it was for Surgeon-General Sternberg to supply the deficiency by employing acting assistant surgeons. Now England has been forced to supply a similar lack in a similar manner, and a large number of civilian surgeons and nurses have been sent to South Africa. The nurses were drawn from the Army Nursing Service Reserve.

The English contract surgeons are paid about the same as American contract surgeons are—£1 a day—but, whereas the American contract surgeon, by one of those elastic rulings of the Treasury department, gets $150 per month and no allowances of any kind whatever, not even sick leave pay, the English surgeon receives about the same amount of money, a horse, and the full allowance of a captain. At the conclusion of their service they receive two months extra pay. The English nurses receive £40 a year, with pay allowances, and £20 gratuity at the end of their service.

The English War Department has also sent three eminent surgeons to South Africa as consultants—MacCormac, Treves and Makins. These men receive $25,000 a year. On the other hand, the United States paid Senn and other consultants $3,000 and gave them commissions as lieutenant-colonels.

There is quite a difference here, in the treatment of surgeons, and a movement is on foot to regulate the status of American acting assistant surgeons. Surgeon-General Sternberg has prepared a bill which will give them all the allowances of a first-lieutenant and all the rights of a commissioned officer. The recently organised association of acting assistant surgeons will also present a bill fixing their status and regulating their pay and allowances.

